# Effects of Clopidogrel on the Anticonvulsant Efficacy of Valproic Acid and Levetiracetam in a PTZ-Induced Seizure Model

**DOI:** 10.3390/ijms27146415

**Published:** 2026-07-19

**Authors:** Sibel Özdemir, Ertugrul Bolayir, Zeynep Deniz Şahin İnan

**Affiliations:** 1 Department of Neurology, Yunus Emre State Hospital, Eskişehir 26140, Türkiye; 2 Department of Neurology, Faculty of Medicine, Cumhuriyet University, Sivas 58140, Türkiye; ebolayir@cumhuriyet.edu.tr; 3 Department of Histology & Embryology, Faculty of Medicine, Cumhuriyet University, Sivas 58140, Türkiye; zinan@cumhuriyet.edu.tr

**Keywords:** epilepsy, clopidogrel, valproic acid, levetiracetam, pentylenetetrazol, pharmacodynamic interaction, antiplatelet therapy

## Abstract

Patients with cerebrovascular disease frequently require concomitant antiplatelet and antiseizure therapy. However, experimental evidence regarding potential pharmacodynamic interactions between these medications remains limited. The present study investigated whether clopidogrel affects the anticonvulsant effects of valproic acid (VPA) or levetiracetam (LEV) in a pentylenetetrazol (PTZ)-induced seizure model. Forty-two male Wistar rats were randomly allocated to seven groups (n = 6/group): Control, PTZ, clopidogrel (CLP), VPA, LEV, VPA + CLP + PTZ, and LEV + CLP + PTZ. Electroencephalographic recordings, passive avoidance testing, histopathological examination, and glial fibrillary acidic protein (GFAP) immunohistochemistry were performed to evaluate seizure activity, behavioral performance, neuronal injury, and astroglial activation. Both VPA and LEV significantly reduced spike–wave discharge frequency and duration compared with the PTZ group (*p* < 0.05). Clopidogrel alone did not exhibit anticonvulsant activity, and co-administration with either VPA or LEV did not significantly alter electrophysiological, behavioral, histopathological, or GFAP-associated immunohistochemical outcomes. Overall, these findings indicate that clopidogrel did not significantly affect the anticonvulsant efficacy of VPA or LEV under the experimental conditions of this acute PTZ-induced seizure model.

## 1. Introduction

Epilepsy is one of the most common chronic neurological disorders, affecting approximately 50 million people worldwide and representing a major cause of disability and reduced quality of life [1]. Although epilepsy may arise from diverse etiologies, cerebrovascular disease is one of the leading causes of acquired epilepsy in adults, particularly in the elderly population [2,3]. Post-stroke epilepsy accounts for a substantial proportion of late-onset epilepsies and has become an increasingly important clinical problem owing to the aging population and improved survival after ischemic stroke [4,5]. The mechanisms underlying epileptogenesis after stroke are complex and involve neuronal injury, neuroinflammation, oxidative stress, blood–brain barrier dysfunction, and maladaptive neuronal network remodeling, ultimately resulting in persistent neuronal hyperexcitability [6,7].

Antiplatelet therapy is the cornerstone of secondary prevention after ischemic stroke, and clopidogrel is one of the most frequently prescribed agents because of its well-established efficacy and favorable safety profile [8,9,10]. Consequently, many patients who develop epilepsy following stroke receive concomitant treatment with antiplatelet and antiseizure medications (ASMs) to reduce the risk of recurrent vascular events while maintaining adequate seizure control [5,11]. Although this therapeutic combination is common in clinical practice, experimental evidence regarding potential pharmacodynamic interactions between clopidogrel and ASMs remains limited [12]. Determining whether clopidogrel influences the anticonvulsant efficacy of commonly prescribed ASMs is therefore clinically relevant, as any interaction could potentially affect treatment outcomes in this growing patient population [12,13].

Clopidogrel irreversibly inhibits platelet P2Y12 receptors, thereby reducing platelet activation and thrombus formation [14]. Despite its widespread clinical use, its potential influence on the anticonvulsant efficacy of commonly prescribed ASMs has not been adequately investigated in experimental studies [12,13].

Valproic acid (VPA) and levetiracetam (LEV) are among the most widely prescribed antiseizure medications for the treatment of both generalized and focal epilepsies and are commonly used in the management of post-stroke epilepsy [5,15]. VPA has long been recognized as a broad-spectrum ASM with established efficacy across multiple seizure types, whereas LEV has gained widespread acceptance because of its favorable tolerability profile, relatively low potential for clinically relevant drug–drug interactions, and ease of use in patients with multiple comorbidities [15,16,17,18]. Nevertheless, despite their widespread clinical use, experimental evidence evaluating whether clopidogrel influences their anticonvulsant efficacy remains scarce [12,13].

Despite the frequent concomitant use of antiplatelet agents and ASMs in patients with cerebrovascular disease, experimental evidence regarding whether clopidogrel alters the anticonvulsant efficacy of commonly prescribed ASMs remains limited. Clarifying this potential interaction is important because it may improve our understanding of concomitant antiplatelet and antiseizure therapy in patients with cerebrovascular disease.

Although post-stroke epilepsy is commonly investigated using ischemic stroke models, such as middle cerebral artery occlusion (MCAO) or photothrombosis, the primary objective of the present study was to evaluate the potential pharmacodynamic interaction between clopidogrel and commonly used antiseizure medications under standardized experimental seizure conditions. Therefore, the PTZ-induced seizure model was selected because it is a well-established and highly reproducible model for evaluating antiseizure drug efficacy and pharmacodynamic interactions [19].

Therefore, the present study aimed to evaluate the effects of clopidogrel on the anticonvulsant efficacy of VPA and LEV in an acute pentylenetetrazol (PTZ)-induced seizure model. Electroencephalographic recordings, passive avoidance testing, histopathological examination, and glial fibrillary acidic protein (GFAP) immunohistochemistry were performed to comprehensively assess seizure activity, behavioral performance, neuronal injury, and astroglial activation. We hypothesized that clopidogrel would not significantly modify the anticonvulsant effects of valproic acid or levetiracetam in the PTZ-induced seizure model.

## 2. Results

### 2.1. Physiological Parameters

Systolic and diastolic blood pressure values were measured before drug administration and on the final day of the experimental protocol. No statistically significant differences were observed in systolic or diastolic blood pressure values either within groups over time or between experimental groups (*p* > 0.05). Because these measurements did not demonstrate meaningful variation and were not directly related to the primary outcomes of the study, the data were not presented in a separate figure or table. These findings indicate that systemic hemodynamic parameters remained stable throughout the experimental period, with no treatment-related differences detected.

### 2.2. Electroencephalographic Findings

Administration of pentylenetetrazol (PTZ) induced marked epileptiform activity, characterized by a significant increase in spike–wave discharge (SWD) frequency and duration compared with the control group (*p* < 0.05) (Table 1).

Treatment with valproic acid (VPA) and levetiracetam (LEV) significantly reduced both SWD frequency and duration compared with the PTZ group (*p* < 0.05). Although VPA-treated groups exhibited numerically greater suppression of SWDs than LEV-treated groups, the difference did not reach statistical significance (*p* > 0.05).

Clopidogrel (CLP) administered alone did not exhibit anticonvulsant effects, as SWD parameters were not significantly different from those of the PTZ group (*p* > 0.05). Co-administration of clopidogrel with either VPA or LEV did not significantly alter SWD parameters compared with the respective antiseizure medications alone (*p* > 0.05).

To further evaluate potential pharmacodynamic interactions, a two-way ANOVA was performed. The analysis demonstrated a significant main effect of ASM treatment on both SWD duration (F = 26.16, *p* < 0.001, η^2^p = 0.567) and SWD frequency (F = 24.82, *p* < 0.001, η^2^p = 0.554). In contrast, neither the main effect of clopidogrel (SWD duration: F = 0.55, *p* = 0.469; SWD frequency: F = 1.51, *p* = 0.233) nor the ASM × clopidogrel interaction (SWD duration: F = 0.005, *p* = 0.942; SWD frequency: F = 0.124, *p* = 0.729) was statistically significant.

### 2.3. Passive Avoidance Test

**Figure 1:**
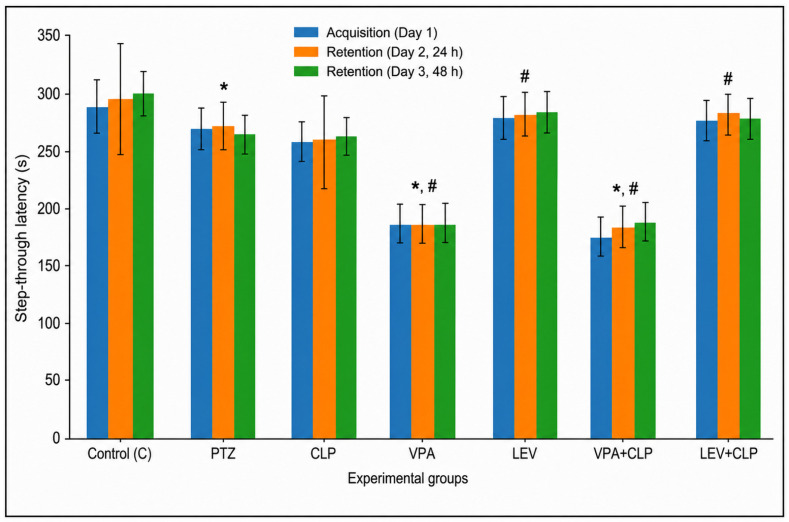
Effects of treatments on passive avoidance performance. Step-through latency values were measured during passive avoidance testing at baseline, 24 h, and 48 h. Blue, orange, and green bars represent baseline, 24 h, and 48 h measurements, respectively. Data are presented as mean ± standard deviation (SD) for six animals per group (n = 6). * *p* < 0.05 vs. control group; # *p* < 0.05 vs. PTZ group. Abbreviations: CLP, clopidogrel; VPA, valproic acid; LEV, levetiracetam.

In the passive avoidance test, animals treated with VPA exhibited significantly shorter step-through latency than control animals during baseline, 24-h, and 48-h retention tests (*p* < 0.05). Comparable findings were observed in the VPA + CLP group (Figure 1).

Repeated-measures ANOVA demonstrated significant main effects of both treatment group and time on step-through latency values (*p* < 0.05), indicating differences among experimental groups and across repeated measurements.

In contrast, LEV-treated animals exhibited latency values comparable to those of the control group (*p* > 0.05). Similarly, the LEV + CLP group showed no significant differences compared with the control group (*p* > 0.05). Clopidogrel administration alone did not significantly affect passive avoidance performance.

No significant ASM × clopidogrel interaction was detected (*p* > 0.05).

### 2.4. Histopathological Findings

**Figure 2:**
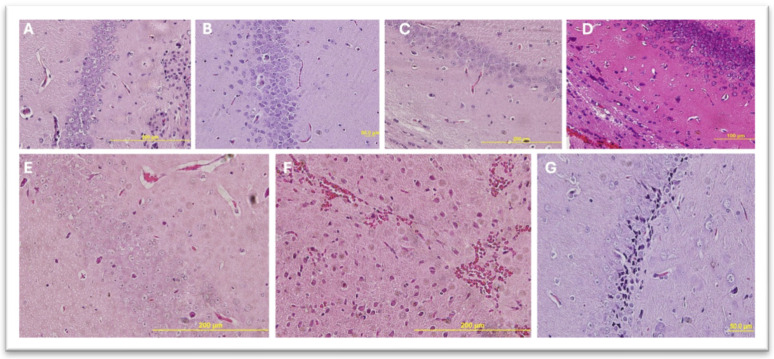
Histopathological changes in hippocampal tissue across experimental groups. Representative hematoxylin–eosin (H&E)-stained hippocampal sections (40× magnification). (**A**) Control group with normal morphology. (**B**) PTZ group showing severe neuronal degeneration, necrosis, inflammation, and numerous red neurons. (**C**) CLP group showing moderate damage. (**D**) VPA group showing reduced neuronal degeneration with preserved hippocampal architecture. (**E**) LEV group showing mild damage with preserved architecture. (**F**) VPA + CLP group showing histological features comparable to the VPA group. (**G**) LEV + CLP group showing histological features comparable to the LEV group.

Histopathological evaluation of hippocampal sections revealed distinct differences among experimental groups with respect to neuronal morphology, necrosis, inflammatory infiltration, and red neuron formation (Figure 2).

The control group exhibited normal hippocampal architecture with well-preserved neuronal morphology and no evidence of necrosis or inflammatory changes. In contrast, the PTZ group showed severe histopathological alterations characterized by widespread neuronal degeneration, prominent necrosis, marked inflammatory infiltration, and numerous red neurons.

The CLP group demonstrated moderate neuronal injury, whereas the VPA- and LEV-treated groups exhibited reduced histopathological damage compared with the PTZ group. Both groups exhibited fewer necrotic neurons, reduced inflammatory infiltration, and fewer red neurons than the PTZ group, with largely preserved hippocampal architecture.

Co-administration of clopidogrel with either VPA or LEV did not result in additional histopathological alterations. Histological findings in the VPA + CLP and LEV + CLP groups were comparable to those observed in the corresponding ASM-treated groups.

**Figure 3:**
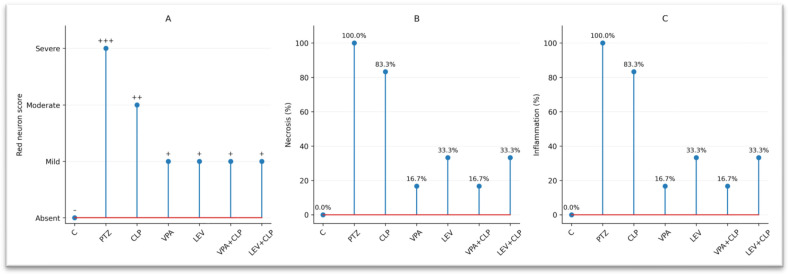
Semi-quantitative analysis of histopathological alterations in hippocampal tissue across experimental groups. (**A**) Red neuron scores indicating the severity of neuronal injury. (**B**) Percentage of animals exhibiting necrosis. GFAP immunoreactivity was semi-quantitatively graded as absent (−), mild (+), moderate (++), or intense (+++). (**C**) Percentage of animals exhibiting inflammatory infiltration. Data are presented as semi-quantitative scores or percentages for six animals per group (n = 6). Abbreviations: CLP, clopidogrel; VPA, valproic acid; LEV, levetiracetam.

Semi-quantitative analysis supported the histopathological observations (Figure 3). The PTZ group exhibited significantly higher red neuron scores and higher rates of necrosis and inflammatory infiltration than the control group (*p* < 0.05). Treatment with VPA and LEV significantly attenuated these pathological changes (*p* < 0.05 vs. PTZ), whereas no significant differences were observed between ASM-treated groups and their corresponding CLP co-treatment groups (*p* > 0.05).

### 2.5. Immunohistochemical Analysis of GFAP

**Figure 4:**
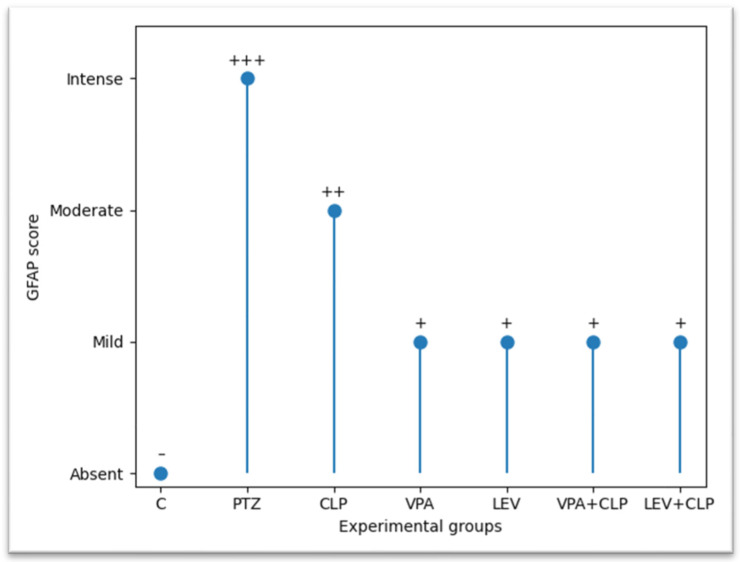
Semi-quantitative analysis of GFAP immunoreactivity in hippocampal tissue. GFAP expression was evaluated using an ordinal scoring system (0–3), corresponding to absent (0), mild (1), moderate (2), and intense (3) staining. Data are presented across experimental groups. GFAP immunoreactivity was semi-quantitatively graded as absent (−), mild (+), moderate (++), or intense (+++). Abbreviations: CLP, clopidogrel; VPA, valproic acid; LEV, levetiracetam.

Immunohistochemical evaluation of hippocampal sections demonstrated marked differences in glial fibrillary acidic protein (GFAP) immunoreactivity among the experimental groups. Semi-quantitative analysis revealed significantly higher GFAP immunoreactivity scores in the PTZ group than in the control group (Figure 4, *p* < 0.05).

The control group exhibited weak GFAP immunostaining with normal astrocytic morphology. In contrast, the PTZ group showed intense GFAP immunoreactivity, characterized by increased astrocyte density and hypertrophic astrocytic processes.

The clopidogrel (CLP) group exhibited mild to moderate GFAP immunoreactivity. Treatment with valproic acid (VPA) and levetiracetam (LEV) was associated with lower GFAP immunoreactivity than that observed in the PTZ group. Similarly, the VPA + CLP and LEV + CLP groups exhibited GFAP immunoreactivity patterns comparable to those of the corresponding ASM-only groups.

**Figure 5:**
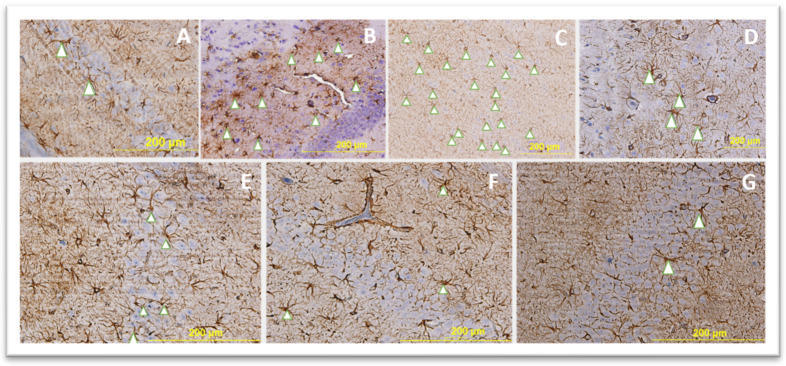
Representative GFAP immunohistochemical staining in hippocampal tissue. Representative photomicrographs of GFAP immunostaining in hippocampal sections from experimental groups (40× magnification). GFAP-positive astrocytes are identified by brown cytoplasmic staining with characteristic cellular processes. (**A**) Control group. (**B**) PTZ group. (**C**) CLP group. (**D**) VPA group. (**E**) LEV group. (**F**) VPA + CLP group. (**G**) LEV + CLP group. Scale bar = 200 µm. White arrows indicate GFAP localization in the hippocampal region.

Representative GFAP immunohistochemical staining patterns for all experimental groups are shown in Figure 5. Consistent with the semi-quantitative analysis, GFAP immunoreactivity was highest in the PTZ group and was significantly lower in the VPA, LEV, VPA + CLP, and LEV + CLP groups than in the PTZ group (*p* < 0.05). No statistically significant differences were observed between the ASM-only groups and their corresponding ASM + CLP groups (*p* > 0.05).

## 3. Discussion

The present electrophysiological, behavioral, histopathological, and immunohistochemical findings collectively demonstrate that clopidogrel did not significantly modify the anticonvulsant effects of valproic acid or levetiracetam in this acute PTZ-induced seizure model. Both valproic acid and levetiracetam significantly suppressed epileptiform activity, whereas clopidogrel neither exhibited anticonvulsant effects nor altered their efficacy. Likewise, co-administration of clopidogrel did not further impair passive avoidance performance, increase neuronal injury, or enhance GFAP immunoreactivity compared with antiseizure medication treatment alone.

The PTZ-induced seizure model is a well-established experimental model for evaluating antiseizure drug activity and remains widely used in preclinical pharmacological studies because of its reliability and reproducibility [20,21]. The model was deliberately selected because the primary objective of the present study was to evaluate potential pharmacodynamic interactions between clopidogrel and commonly used antiseizure medications under standardized experimental seizure conditions rather than to investigate the mechanisms of post-stroke epileptogenesis. In contrast, ischemic stroke models such as middle cerebral artery occlusion (MCAO) or photothrombosis are primarily designed to investigate the mechanisms of post-stroke epileptogenesis following cerebral ischemia and are therefore less suitable for standardized pharmacodynamic interaction studies [19]. However, because PTZ induces acute chemically evoked seizures, this model does not fully reproduce the complex pathophysiological mechanisms underlying post-stroke epilepsy or chronic epileptogenesis. Therefore, the findings of the present study should be interpreted within the context of acute PTZ-induced epileptiform activity, and extrapolation to post-stroke epilepsy, post-traumatic epilepsy, or chronic epileptic disorders should be made with caution. In the present study, both valproic acid and levetiracetam significantly reduced spike–wave discharge frequency and duration, consistent with previous experimental studies demonstrating their efficacy in PTZ-induced seizure models [22].

Although valproic acid produced numerically greater suppression of epileptiform activity than levetiracetam, it was also associated with shorter step-through latency in the passive avoidance test. These findings should be interpreted with caution, as reduced latency may reflect not only memory impairment but also the sedative and motor effects associated with valproic acid. Previous experimental and clinical studies have reported that valproic acid may influence behavioral performance through dose-dependent sedative and cognitive effects independent of its antiseizure efficacy [23,24]. The dose used in the present study was within the range commonly employed in PTZ-induced seizure models; however, a contribution of sedative effects to the observed behavioral changes cannot be excluded. In contrast, levetiracetam preserved passive avoidance performance while maintaining significant antiseizure efficacy, consistent with previous reports demonstrating its favorable neurobehavioral profile [17]. These findings highlight the importance of considering behavioral outcomes together with anticonvulsant efficacy when interpreting treatment responses in experimental seizure models.

A central aim of this study was to determine whether clopidogrel modifies the anticonvulsant effects of commonly used antiseizure medications. Although clopidogrel primarily exerts its antiplatelet activity through irreversible inhibition of platelet P2Y12 receptors, these receptors are also expressed by microglia within the central nervous system, where they are involved in neuroinflammatory responses associated with seizure generation [24,25,26]. Therefore, modulation of P2Y12 signaling could theoretically influence seizure susceptibility or alter the efficacy of antiseizure medications. However, no evidence of such a pharmacodynamic interaction was observed in the present study. Clopidogrel did not significantly modify the anticonvulsant effects of either valproic acid or levetiracetam across electrophysiological, behavioral, histopathological, and immunohistochemical assessments. Clinically, this finding may be relevant because patients with cerebrovascular disease frequently require concomitant antiplatelet and antiseizure therapy, particularly following ischemic stroke [25,26,27]. The concordance between the electrophysiological, behavioral, histopathological, and immunohistochemical findings further strengthens the overall interpretation of the present results.

Histopathological and immunohistochemical findings were in agreement with the electrophysiological results. PTZ-induced seizures produced marked neuronal degeneration, necrosis, inflammatory infiltration, and increased GFAP immunoreactivity, consistent with seizure-associated neuronal injury and reactive astrogliosis [28,29].

A major strength of the present study is the integration of electrophysiological, behavioral, histopathological, and immunohistochemical assessments, allowing a comprehensive evaluation of the potential interaction between clopidogrel and commonly used antiseizure medications. The use of these complementary methodologies provided a multidimensional assessment of seizure-related outcomes rather than relying on a single experimental endpoint, thereby increasing confidence in the overall interpretation of the results.

Several limitations of the present study should be acknowledged. First, this study employed an acute PTZ-induced seizure model; therefore, the findings should be interpreted within the context of acute seizure activity and may not fully reflect chronic epilepsy, epileptogenesis, or post-stroke epilepsy. Second, the sample size was relatively modest, and plasma or brain drug concentrations were not measured; therefore, potential pharmacokinetic interactions between clopidogrel and the investigated antiseizure medications could not be evaluated. Third, only male rats were included, precluding assessment of potential sex-related differences in seizure susceptibility, neuroinflammatory responses, and drug interactions.

The study primarily focused on astroglial activation using GFAP immunohistochemistry. Although histopathological and immunohistochemical evaluations were performed using blinded semi-quantitative scoring, quantitative digital image analysis, region-specific hippocampal assessment, and additional neuroinflammatory markers such as microglial markers and inflammatory cytokines were not evaluated. Consequently, mechanistic conclusions regarding seizure-related neuroinflammatory pathways should be interpreted with caution.

Electrophysiological assessment was based primarily on spike–wave discharge frequency and duration. Additional EEG parameters, including interictal spikes, sharp waves, spectral EEG analysis, behavioral seizure severity (Racine score), and simultaneous video-EEG monitoring, were not included. Therefore, the electrophysiological findings should be interpreted as indicators of PTZ-induced epileptiform activity rather than a comprehensive characterization of seizure severity.

Finally, hematological parameters, platelet function, and bleeding-related outcomes were not evaluated, despite their potential relevance to clopidogrel therapy, particularly in combination with valproic acid. Future studies using chronic epilepsy models, quantitative histological analyses, comprehensive neuroinflammatory profiling, and hematological safety assessments are warranted to further clarify the interaction between clopidogrel and antiseizure medications. Despite these limitations, the present study provides a comprehensive experimental evaluation of the potential pharmacodynamic interaction between clopidogrel and two commonly used antiseizure medications using complementary electrophysiological, behavioral, histopathological, and immunohistochemical approaches.

## 4. Materials and Methods

### 4.1. Ethical Approval and Animals

This study was conducted in accordance with international guidelines for the care and use of laboratory animals and complied with the ARRIVE guidelines [29]. The experimental protocol was approved by the Cumhuriyet University Faculty of Medicine Animal Experiments Local Ethics Committee (Approval No: B.30.2.CUM.0.01.00.00-50/106).

A total of 42 adult (10–12-week-old) male Wistar albino rats weighing 250–275 g were obtained from the Experimental Animal Research and Application Center of Cumhuriyet University, where they were bred and maintained under controlled laboratory conditions. Animals were housed under controlled environmental conditions (22 ± 2 °C, 55 ± 10% humidity, 12 h light/dark cycle) with ad libitum access to food and water. Animals were acclimatised to the housing conditions for at least 7 days before the start of the experimental procedures. All procedures were designed to minimize animal suffering. No unexpected adverse events, procedure-related complications, or animal losses occurred during the study. All animals were experimentally naïve, clinically healthy, and had not undergone any previous experimental procedures. No genetic modifications were present.

No predefined humane endpoints were established because no animals developed severe or persistent distress requiring early euthanasia during the study.

### 4.2. Experimental Design and Drug Administration

Animals were randomly assigned into seven groups (n = 6 per group): Control, PTZ, clopidogrel (Sigma-Aldrich, St. Louis, MO, USA) (CLP), valproic acid (Sigma-Aldrich, St. Louis, MO, USA) (VPA), levetiracetam (Sigma-Aldrich, St. Louis, MO, USA) (LEV), VPA + CLP, and LEV + CLP. Animals were randomly assigned to groups on a per-animal basis.

Clopidogrel was administered by oral gavage at a dose of 10 mg/kg/day for six consecutive days [13]. Valproic acid (300 mg/kg/day) [15] and levetiracetam (80 mg/kg/day) [16] were administered intraperitoneally for six consecutive days according to previously published experimental protocols.

Control animals received equivalent volumes of physiological saline. On day 6, pentylenetetrazol Sigma-Aldrich, St. Louis, MO, USA) (PTZ, 60 mg/kg, i.p.) was administered to all experimental groups except the Control group to induce seizures, after which electroencephalographic recordings, behavioral testing, histopathological evaluation, and immunohistochemical analyses were performed [20].

**Figure 6:**
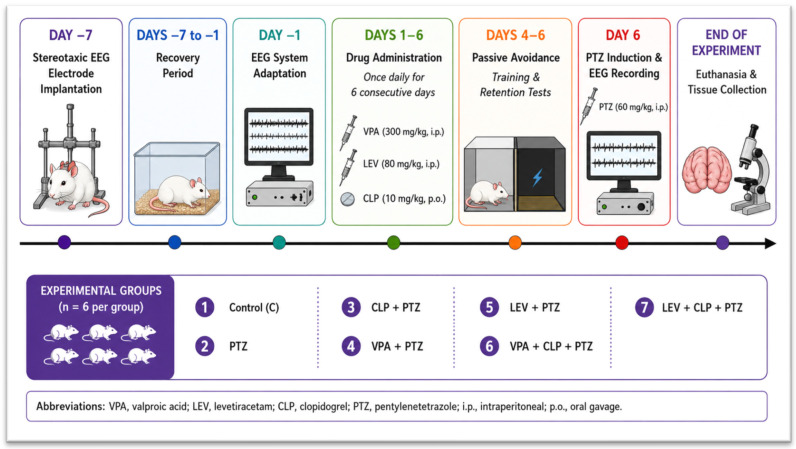
Experimental design and study timeline. Schematic overview of the experimental protocol, illustrating stereotaxic EEG electrode implantation, postoperative recovery, EEG system adaptation, drug administration, passive avoidance testing, PTZ-induced seizure induction, EEG recording, and subsequent histopathological and immunohistochemical analyses.

The overall experimental workflow is summarized in Figure 6.

### 4.3. Stereotaxic Surgery and EEG Recording

All animals underwent stereotaxic surgery 7 days before seizure induction. Anesthesia was induced with ketamine (80 mg/kg, i.p.; Ketalar®, Pfizer Inc., New York, NY, USA) and xylazine (10 mg/kg, i.p.; Rompun®, Bayer AG, Leverkusen, Germany), after which the animals were positioned in a stereotaxic frame. Four stainless steel screw electrodes were implanted over the cortical surface according to stereotaxic coordinates derived from the rat brain atlas [20]. The electrodes were connected to a miniature connector system and secured to the skull using dental acrylic. Following surgery, animals were allowed a 7-day recovery period before seizure induction to minimize postoperative stress and ensure complete recovery.

Following a 7-day recovery period, electroencephalographic (EEG; David Kopf Instruments, Tujunga, CA, USA) recordings were obtained using a multichannel recording system while the animals were freely moving. EEG signals were amplified, filtered (0.1–100 Hz), and digitally recorded for subsequent analysis. Baseline EEG activity was recorded for 2 h after drug administration. Following PTZ administration, EEG recordings were continued for an additional 2 h. Each animal underwent a single acute EEG recording session, and spike–wave discharge (SWD) frequency and duration were analyzed as the primary electrophysiological outcomes. The primary outcome measure of the study was the frequency and duration of spike–wave discharges (SWDs) recorded by EEG, which served as the principal endpoint for evaluating the anticonvulsant effects of the treatments.

**Figure 7:**
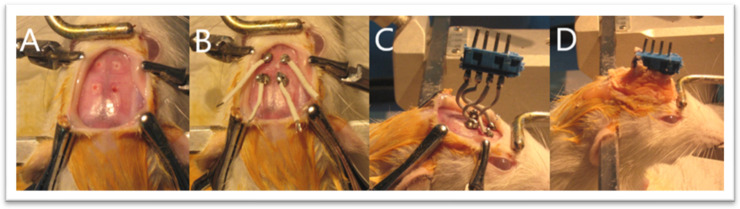
Stereotaxic implantation of cortical EEG electrodes in rats. (**A**) Exposure of the skull following a midline incision under anesthesia. (**B**) Placement of stainless steel screw electrodes over the frontal and parietal cortices according to stereotaxic coordinates based on the Paxinos and Watson rat brain atlas. (**C**) Connection of electrodes to a miniature connector system. (**D**) Final fixation of the electrode assembly using dental acrylic.

**Figure 8:**
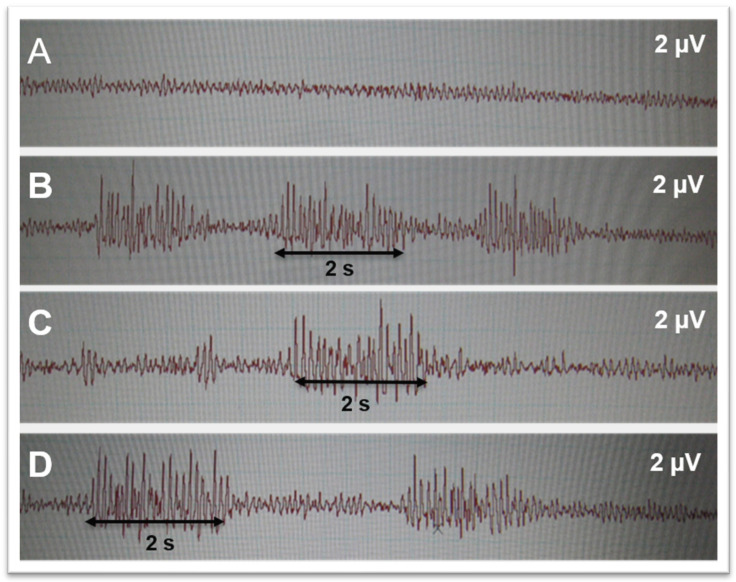
Representative electroencephalographic (EEG) recordings obtained from the experimental groups. (**A**) Control group showing normal baseline cortical activity without spike–wave discharges (SWDs). (**B**) PTZ-treated group demonstrating representative SWD activity (2 s). (**C**) Valproic acid (VPA)-treated group showing reduced SWD activity. (**D**) Levetiracetam (LEV)-treated group showing attenuation of SWD activity. Vertical scale: 2 µV.

The electrode implantation procedure is illustrated in Figure 7, and representative EEG recordings are presented in Figure 8.

All EEG recordings were analyzed by an investigator blinded to the experimental groups to minimize observer bias.

### 4.4. Spike–Wave Discharge Analysis

EEG recordings were analyzed both visually and quantitatively for spike–wave discharges (SWDs). Only spike–wave complexes lasting ≥2 s were included in the analysis (Figure 8), consistent with established criteria for experimental PTZ-induced seizure activity [19,30,31]. SWDs were identified offline by visual inspection.

For each animal, SWD frequency and total SWD duration were calculated from EEG recordings obtained following PTZ administration. All EEG analyses were performed by an investigator blinded to the experimental groups to minimize observer bias.

### 4.5. Passive Avoidance Test

Cognitive performance was assessed using the passive avoidance test, a widely used behavioral paradigm for evaluating learning and memory in experimental epilepsy models. The apparatus consisted of two compartments—one illuminated and one dark—separated by a sliding door (Panlab, Barcelona, Spain).

Prior to training, animals were allowed to explore both compartments during a brief habituation period. During the training session, each animal was placed in the illuminated compartment and received a mild foot shock (1 mA for 2 s) upon entering the dark compartment. Acquisition trials were performed before PTZ administration to establish baseline learning performance, whereas retention tests were conducted at 24 and 48 h following PTZ-induced seizure induction.

Memory retention was assessed by measuring the step-through latency to enter the dark compartment. No electrical stimulation was delivered during retention testing. A cut-off time of 300 s was applied, and animals that did not enter the dark compartment within this period were assigned the maximum latency value.

### 4.6. Histopathological and Immunohistochemical Evaluation

At the end of the experimental protocol, animals were euthanized under deep anesthesia, and brain tissues were rapidly removed. Samples were fixed in 10% neutral-buffered formalin (Thermo Fisher Scientific, Waltham, MA, USA), embedded in paraffin, and sectioned at 4–5 µm thickness.

Hematoxylin–eosin (H&E) staining was performed to evaluate neuronal morphology, necrosis, inflammatory infiltration, and red neuron formation using coronal sections containing the hippocampal formation as a whole, rather than separate quantitative analyses of specific subregions such as CA1, CA3, or the dentate gyrus. Histopathological assessment was conducted using a semi-quantitative scoring approach based on established criteria [28]. Histopathological and immunohistochemical scoring was performed by an investigator blinded to the experimental groups to minimize observer bias.

Red neurons were scored semi-quantitatively as mild (+, 0–10 cells), moderate (++, 10–20 cells), or severe (+++, >20 cells) within representative hippocampal microscopic fields selected from coronal sections containing the hippocampal formation under 40× magnification using a light microscope (Olympus BX53, Olympus Corporation, Tokyo, Japan). At least five non-overlapping microscopic fields were evaluated from each section.

For immunohistochemical analysis, glial fibrillary acidic protein (GFAP) expression was evaluated as a marker of astroglial activation. Following deparaffinization and rehydration, antigen retrieval was performed using citrate buffer (pH 6.0; Thermo Fisher Scientific, Waltham, MA, USA) at 95 °C. Endogenous peroxidase activity was blocked with 3% hydrogen peroxide (Merck, Darmstadt, Germany), and nonspecific binding was prevented using an appropriate protein blocking solution (Thermo Fisher Scientific, Waltham, MA, USA). Sections were incubated with primary anti-GFAP antibody (Abcam, Cambridge, UK), followed by a suitable secondary antibody (Abcam, Cambridge, UK), and visualized using a diaminobenzidine (DAB) chromogen system (Thermo Fisher Scientific, Waltham, MA, USA).

GFAP immunoreactivity was assessed in five non-overlapping microscopic fields per section and semi-quantitatively scored as absent (0), mild (+), moderate (++), or intense (+++) according to staining intensity using previously established semi-quantitative criteria [28]. Digital image-based quantification of GFAP-positive cell counts or staining intensity was not performed.

All histopathological and immunohistochemical evaluations were performed independently by two histologists who were blinded to the experimental groups. In cases of disagreement, the slides were jointly re-evaluated until a consensus was reached.

### 4.7. Statistical Analysis

Statistical analyses were performed using IBM SPSS Statistics for Windows (version 20.0; IBM Corp., Armonk, NY, USA). Data distribution was assessed using the Shapiro–Wilk test, and homogeneity of variances was evaluated using Levene’s test. When the assumptions for parametric analyses were not met, appropriate non-parametric tests were applied.

For electroencephalographic variables, including spike–wave discharge (SWD) duration and frequency, group comparisons were performed using one-way analysis of variance (ANOVA) followed by Tukey’s post hoc test.

To evaluate whether clopidogrel modifies the anticonvulsant efficacy of antiseizure medications, a two-way ANOVA was conducted with ASM treatment (valproic acid vs. levetiracetam) and clopidogrel administration (present vs. absent) as fixed factors. Interaction effects (ASM × clopidogrel) were assessed, and effect sizes were reported as partial eta squared (η^2^p).

Behavioral data from the passive avoidance test were analyzed using mixed-design two-way repeated-measures ANOVA, with time (baseline, 24 h, and 48 h) as the within-subject factor and treatment group as the between-subject factor. When significant effects were observed, post hoc comparisons were performed using Tukey’s test.

Categorical variables, including necrosis and inflammatory infiltration, were analyzed using Fisher’s exact test. Ordinal variables, including red neuron scores and GFAP immunoreactivity, were analyzed using the Kruskal–Wallis test followed by Dunn’s multiple-comparisons post hoc test.

All statistical tests were two-tailed, and a *p* value < 0.05 was considered statistically significant.

### 4.8. Sample Size and Power Analysis

An a priori sample size calculation was performed using G*Power software (version 3.1) for a one-way ANOVA with seven experimental groups, assuming a large effect size (Cohen’s f = 0.80), a significance level (α) of 0.05, and a statistical power (1 − β) of 0.80. The minimum required total sample size was calculated to be 28 animals.

To increase the robustness of the statistical analyses, a total of 42 rats (n = 6 per group) were included in the study. A post hoc power analysis confirmed that the achieved sample size provided adequate statistical power for the primary outcome comparisons.

## 5. Conclusions

The present study demonstrated that clopidogrel did not significantly modify the anticonvulsant efficacy of valproic acid or levetiracetam in the PTZ-induced seizure model. Furthermore, co-administration of clopidogrel did not further impair passive avoidance performance, increase histopathological alterations, or enhance GFAP-associated astroglial activation.

These findings suggest that clopidogrel does not substantially influence PTZ-induced epileptiform activity, histopathological alterations, or GFAP-associated astroglial responses during treatment with valproic acid or levetiracetam in this acute experimental model. However, because microglial activation and direct P2Y12-mediated neuroinflammatory pathways were not specifically evaluated, potential microglia-related effects of clopidogrel cannot be excluded.

Because the present study employed an acute PTZ-induced seizure model, further studies using chronic epilepsy models together with direct assessments of microglial signaling are warranted to better define the potential neuroinflammatory effects of clopidogrel in epilepsy.

## Figures and Tables

**Table 1:** Effects of treatments on spike–wave discharge (SWD) duration and frequency.

Group	SWD Duration (s)	SWD Frequency (n)
C	0.00 ± 0.00	0.00 ± 0.00
PTZ	232.39 ± 10.15 *	114.67 ± 6.83 *
CLP	227.51 ± 18.75 *	109.00 ± 8.15 *
VPA	74.72 ± 9.40 #	43.17 ± 7.70 #
LEV	94.09 ± 6.70 #	56.33 ± 6.22 #
VPA + CLP	77.75 ± 8.18 #	45.67 ± 7.55 #
LEV + CLP	96.58 ± 10.47 #	60.83 ± 6.18 #

Data are presented as mean ± standard deviation (SD). Statistical comparisons were performed using one-way analysis of variance (ANOVA) followed by Tukey’s post hoc test. In addition, two-way ANOVA was used to evaluate the interaction between ASMs and clopidogrel. SWD frequency is expressed as the number of spike–wave discharge events per minute (events/min). * *p* < 0.05 vs. control group; # *p* < 0.05 vs. PTZ group. Abbreviations: C, control; PTZ, pentylenetetrazol; CLP, clopidogrel; VPA, valproic acid; LEV, levetiracetam.

## Data Availability

The datasets generated and/or analyzed during the current study are available from the corresponding author on reasonable request.

## Abbreviations

ASM	Antiseizure medication
CLP	Clopidogrel
EEG	Electroencephalography
GFAP	Glial fibrillary acidic protein
LEV	Levetiracetam
PTZ	Pentylenetetrazol
SWD	Spike–wave discharge
VPA	Valproic acid
